# The Influence of Physical Training on the Immune System of Rats during N-methyl-N-nitrosourea-Induced Carcinogenesis

**DOI:** 10.3390/jcm11216371

**Published:** 2022-10-28

**Authors:** Iwona Malicka, Katarzyna Siewierska, Mateusz Olbromski, Natalia Glatzel-Plucinska, Marzenna Podhorska-Okolow, Piotr Dziegiel, Marek Wozniewski

**Affiliations:** 1 Department of Physiotherapy, Wroclaw University of Health and Sport Sciences, 51-612 Wroclaw, Poland; 2 Division of Histology and Embryology, Department of Human Morphology and Embryology, Wroclaw Medical University, 50-368 Wroclaw, Poland

**Keywords:** breast cancer, lymphocyte subpopulations, N-methyl-N-nitrosourea, physical training

## Abstract

Aim: To assess the effect of physical training on the selected parameters of the immune system regarding CD3, CD4, CD8, CD11, CD161, CD45A cell counts in rats treated with N-methyl-N-nitrosourea (MNU). Material and Methods: Thirty-eight female Sprague-Dawley rats were injected intraperitoneally with MNU and were divided into three groups, i.e., sedentary control (SC), the group of moderate-intensity training (MIT) and the group of high-intensity training (HIT). Physical training was supervised immediately after MNU administration and was conducted 5 days per week for 12 weeks on a three-position treadmill. Results: A significant difference was found between SC and training groups in terms of the number of induced tumors per rat (1.57 vs. 0.4, *p* = 0.05) and in the following lymphocyte subpopulations: CD4+/CD8+ (*p* = 0.01), CD3−/CD11b+ (*p* = 0.02), CD3−/CD161+ (*p* = 0.002), CD3−/CD161− (*p* = 0.002), CD3+/CD45RA+ (*p* = 0.003) and CD3−/CD45RA+ (*p* = 0.005). In terms of the intensity of physical training, the highest efficacy was found for MIT and the following lymphocyte subpopulations: CD3−/CD11b+ (SC vs. MIT, *p* < 0.001), CD3−/CD161+ (SC vs. MIT, *p* = 0.002), CD3−/CD161− (SC vs. MIT, *p* = 0.002), CD3+/CD45RA+ (SC vs. MIT, *p* = 0.02) and CD3−/CD45RA+ (SC vs. MIT, *p* < 0.001, MIT vs. HIT, *p* = 0.02). Furthermore, negative correlations were found between the number of apoptotic cells and CD3−/CD11b (r = −0.76, *p* = 0.01) in SC and between the number of induced tumors and CD3+/CD8+ (r = −0.61, *p* = 0.02) and between their volume and CD+/CD8+ (r = −0.56, *p* = 0.03) in the group of rats undergoing training. Conclusions: Physical training, particularly MIT, affected immune cell function and an altered immune response can be considered a mechanism underlying the effect of exercise on breast cancer development.

## 1. Introduction

Breast cancer is the most prevalent malignancy in women worldwide. More than 2.3 million new cases were diagnosed in 2018 [1]. Due to an estimated further increase in the incidence of breast cancer and an increase in patient survival, a group of women undergoingtreatment for breast cancer will constitute a significant and growing population in the coming years.

An important role in the process of tumor growth is attributed to CD4+ and CD8+ lymphocytes and the cytokines secreted by these cells. A review of the literature clearly shows that CD4+ cells are predominant among lymphocytes infiltrating the tumor [2,3]. The CD4+/CD8+ ratio plays a prognostic role [2,4]. Studies have confirmed that, in breast cancer, tumors are infiltrated by a heterogeneous immune cell population, including T-cells, B-cells, natural killer (NK) cells and macrophages. Tumor-infiltrating lymphocytes, which are a primary immune component infiltrating solid tumors, are regarded as the manifestation of the host’s antitumor reaction. Most tumor-infiltrating lymphocytes in solid tumors are of the CD3+ T-cell phenotype, including CD4+ helper cells, CD4+ regulatory T-cells and CD8+cytotoxic T lymphocytes (CTLs). Due to triggering apoptosis, CTLs destroy tumor cells. As a result, they may exert an effect on tumor growth [5].

At the same time, the importance of physical activity is stressed in both the primary and secondary prevention of breast cancer. For women whose physical activity was insufficient before breast cancer diagnosis, but in whom physical activity was increased after diagnosis to the recommended levels of 7.5 MET-h/week, the following were reported: a significant 50% reduction in overall mortality, a 46% reduction in breast cancer mortality, and a 42% improvement in recurrence-free survival compared to women whose physical activity was insufficient [6].

Improvement in immune functioning is one of the factors that are considered protective and modifiable in terms of physical activity [6,7,8]. Exercise -induced immunomodulation is related to the magnitude of physical load. Depending on the exercise intensity and duration, physical activity can induce different immune responses [9]. It is confirmed that MIT stimulates immune mechanisms and has a protective effect [10,11], whereas the study results are not conclusive for HIT. On the one hand, HIT can lead to immunosuppression. Animal experiments showed that such suppression resulted not only in increased susceptibility to respiratory tract infections, but also in increased mortality due to these infections [12]. One experimental study also showed a higher risk of cancer development and metastasis in rats undergoing exhaustive physical exercise [13]. On the other hand, the activation of glutamine metabolism in macrophages and lymphocytes induced by the presence of Walker 256 tumor was prevented by high-intensity training, increasing the lifespan of rats and reducing tumor size [14].

Since rats and mice have breast glands that are similar to human glands in terms of structure and function, they are the main species that undergo experimental studies on breast cancer [15]. In turn, N-methyl N-nitrosourea (MNU), which is a recognized carcinogen inducing tumorigenesis of breast cancer, is commonly used in such studies [16,17]. Such studies also use supervised physical training on the treadmill or physical activity (i.e., voluntary wheel running). Treadmill is a common exercise intervention applied in most studies. The advantage of treadmill training is that the exercise intensity can be controlled artificially by regulating the speed or slope. As opposed to the treadmill training, the activity intensity and duration of voluntary wheel-running cannot be controlled. Swimming or resistance training can also be used [18].

The aim of this study was to assess the effect of moderate- and high-intensity physical training on the selected immune system parameters regarding CD3, CD4, CD8, CD11, CD161, CD45A cell counts in MNU-treated rats.

## 2. Materials and Methods

### 2.1. Animals

Thirty-eight female Sprague-Dawley rats aged 28 days obtained from the Center for Experimental Medicine (Medical University of Silesia, Katowice, Poland) underwent assessment at the Animal Research Facility in the Department of Pathomorphology (Wroclaw Medical University). Stable living conditions were provided to all animals (constant temperature and humidity, 12/12 h light and dark cycle, rat chow and water ad libitum). The research procedures were conducted in accordance with the approval of the Local Ethics Committee for Animal Experiments (no. 37/2010).

Inclusion criterion:

Completion of 6 out of 8 weeks (75%) of physical training in which the speed and the duration of training were increased, and the following 4 weeks (100%) of training with the constant speed and duration of training (Table 1).

### 2.2. Intoxication with MNU

After a 2-week quarantine period, the animals were intraperitoneally injected with MNU (Sigma-Aldrich, Munich, Germany) at a dose of 180 mg/kg. Next, the animals were monitored in accordance with the previously reported procedure [17,19].

### 2.3. Physical Training

The animals were divided into 2 groups, i.e., the sedentary control (SC) (*n* = 14) and a training group (*n* = 24). Physical training was supervised immediately after MNU administration and was conducted 5 days per week for 12 weeks on a 3-positition treadmill (Exer 3/6; Columbus Instruments, Columbus, OH, USA). Two levels of training intensity were considered, i.e., MIT (*n* = 12) and HIT (*n* = 12), which were adjusted by the speed of the treadmill and training duration (Table 1) [20].

**Table 1:** Physical training protocol [20].

Training [Weeks]	Moderate-Intensity Training		High-Intensity Training	
	Speed of the Treadmill [km/h]	Duration [min.]	Speed of the Treadmill [km/h]	Duration [min.]
1	0.60	10	0.72	12
2	0.96	20	1.15	24
3	1.20	30	1.44	36
4	1.44	40	1.73	48
5	1.68	50	2.02	60
6	1.68	55	2.02	66
7	1.68	60	2.02	72
8	1.68	65	2.02	78
9–12	1.68	30	2.02	36

### 2.4. Tissue Collection

Twelve weeks after MNU administration, the animals were sacrificed by intraperitoneal administration of pentobarbital (200 mg/kg), ketamine (60 mg/kg) and medetomidine (0.5 mg/kg). Tumors detected by palpation were collected and measured and all tissues were fixed in buffered formalin (4%), dehydrated and embedded in paraffin. The blood was also centrifuged (3000 rpm, 15 min. at 4 °C). The obtained plasma was frozen.

### 2.5. Flow Cytometry

Flow cytometry was used to determine immune system cells. The samples were stored on ice all the time. A total of 150 µL of 1% BSA was added to 50 µL of rat blood and was stirred thoroughly. Three mixtures of labelled antibodies were prepared:

1. CD3 APC (557030, Becton Dickinson, Belgium) (5 µL/sample), CD4 FITC (554843, Becton Dickinson, Belgium) (5 µL/sample), CD8a PerCP (558824, Becton Dickinson, Belgium) (5 µL/sample) and CD11b/c PE (554862, Becton Dickinson, Belgium) (5 µL/sample)
2. CD161 FITC (555008, Becton Dickinson, Belgium) (10 µL/sample), CD3 APC (5 µL/sample) and CD45A PE (551402, Becton Dickinson, Belgium) (5 µL/sample)
3. isoFITC (5555743) (5 µL/sample), isoAPC (555745) (5 µL/sample), isoPerCP (559425) (5 µL/sample) and isoPE (555574) (5 µL/sample).

A total of 20 µL of antibody mixture was added per sample and mixed thoroughly. The samples were incubated for 30 min. in the dark. Next, 1 mL of lysis buffer was added and incubated again in the dark for 10 min. The samples were centrifuged at 3000 rpm for 2 min. The supernatant was removed and 1 mL of 1% BSA was added to each sample. The cells were resuspended in the buffer and centrifuged at 3000 rpm. The supernatant was removed again and 1 mL of 1% BSA was added to wash the cells and centrifuged at 3000 rpm for 2 min. After centrifugation, the supernatant was removed. Finally, 400 µL of 1% BSA was added, and the cells were thoroughly resuspended in the solution. The samples were measured using a BD Canto II cytometer (Becton Dickinson). The lymphocyte subpopulations that underwent assessment are given in Table 2.

### 2.6. Tissue Microarrays (TMAs)

Tissue microarrays (TMAs) were prepared after histological examination of tumors, according to the previously described procedure [15]. Using a Manual Tissue Arrayer I (Beecher Instruments Inc., Sun Prairie, WI, USA), triplicate tissue sections (2 mm) with potentially the highest tumor cell content were obtained and transferred to paraffin blocks. The same procedure was repeated for each tumor sample.

### 2.7. Immunohistochemistry (IHC)

Immunohistochemical reactions were performed on 4-μm-thick paraffin sections. The sections were boiled in low pH Target Retrieval Solution (Ki-67 antibody) in PTLink for 20 min. at 97 °C and then cooled in Wash Buffer (TBS buffer with Tween 20) for deparaffinization, rehydration and exposure of antigenic determinants. An EnVision FLEX+ (DakoCytomation, Glostrup, Denmark, cat. no. K8002) was used to perform IHC (AutostainerLink 48). The entire process was consistent with a previously applied procedure [19,21].

### 2.8. TUNEL

To detect apoptotic cells in tumors, the ApopTag In Situ Apoptosis Detection Kit was used (Millipore, cat. no. S7100). The study was performed on 4 µm thick paraffin sections of tumors in accordance with the previous procedure [19,21].

### 2.9. Assessment of IHC

Immunohistochemical expression of Ki-67 antigen and apoptotic cells was assessed in tumors using a BX41 optical microscope equipped with CellD, which is a computer-assisted image analysis software (Olympus Tokyo, Japan). Three measurements from the areas of the most intense expression (‘hot spots’) were selected to assess Ki-67 antigen expression and apoptotic cells in TMAs. The percentage of positive cells in relation to non-expressing cells from each area was assessed by evaluating the brown-labelled cell nuclei of the tumor at 400× magnification. The results obtained from the three samples were recorded as the mean for each tumor.

### 2.10. Statistical Analysis

Statistica 13 (Statsoft, Cracow, Poland) and Prism 5.0 (GraphPad, La Jolla, CA, USA) were used for statistical calculations. The Shapiro–Wilk test was used to check the normality of the distribution, while the Leven test was applied for homogeneity of variance. Descriptive statistics were used, and non-parametric analysis was performed, i.e., the Mann–Whitney U test (SC vs. training groups) and the Kruskal–Wallis ANOVA test to compare three independent groups with the comparison of mean ranks for all samples (post hoc). The correlation between the selected variables was assessed by the Spearman rank coefficient. The analyses were considered statistically significant at *p* ≤ 0.05.

## 3. Results

In the training group, two rats died during the experiment (cause unknown, non-cancerous) and five did not complete the physical training (paw injury). Therefore, the analysis was based on 31 rats, while 10 rats remained in the MIT group and 7 rats remained in the HIT group.

**Figure 1:**
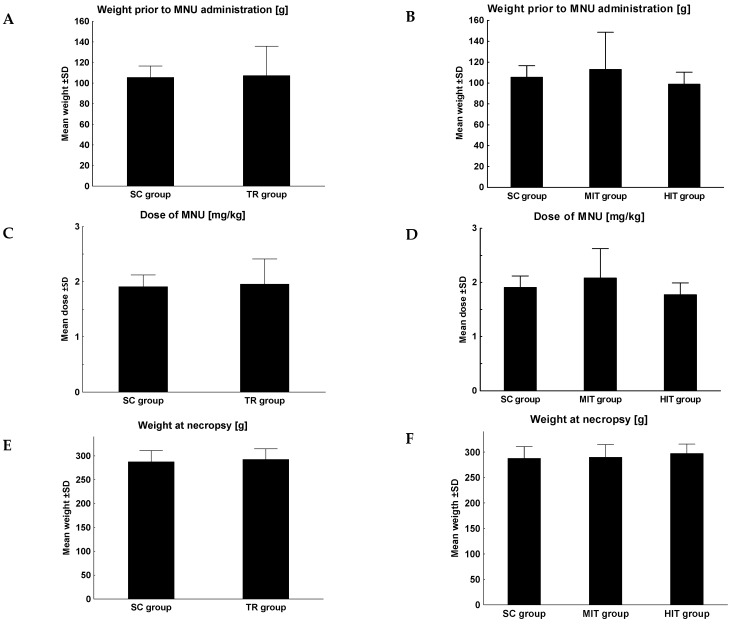

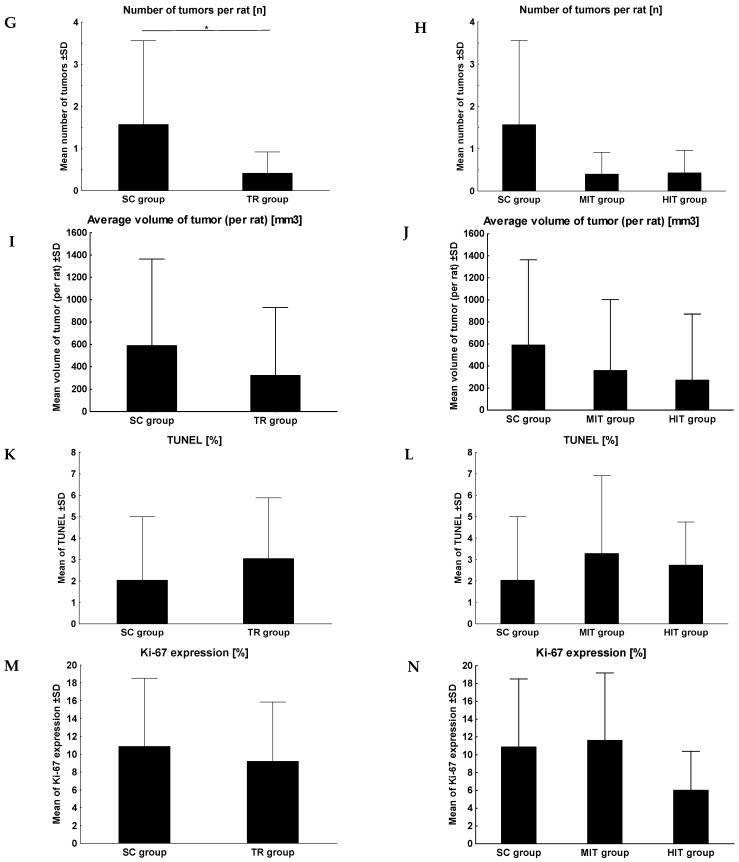
Clinicopathological parameters of the animals included body weight at the time of MNU administration (**A**,**B**), the dose of MNU (**C**,**D**), body weight at necropsy (**E**,**F**), number of tumors per rat (**G**,**H**), tumor volume (**I**,**J**), number of apoptotic cells (**K**,**L**) and the intensity of Ki-67 proliferation (**M**,**N**) in the study groups. Data were analyzed using the Mann–Whitney U test to compare the sedentary group (SC, *n* = 14) with the combined training groups (TR = MIT + HIT, *n* = 24) and the Kruskal–Wallis test was applied for the analysis in particular groups (SC, *n* = 14, MIT, *n* = 12, HIT, *n* = 12). *—the level of divergence between groups (*p* < 0.05); Mann–Whitney U test.

The rats did not differ significantly in terms of body weight or the amount of administered MNU (Figure 1A–F). A significant difference was found in the number of induced tumors per rat between SC and the training group (1.57 vs. 0.4, *p* = 0.05, Figure 1G). However, no significant difference was observed with respect to the level of intensity of physical training (Figure 1H). No significant difference was reported in terms of the tumor volume (Figure 1I,J), the number of apoptotic cells (Figure 1K,L) or the intensity of cell proliferation (Figure 1M,N).

**Figure 2:**
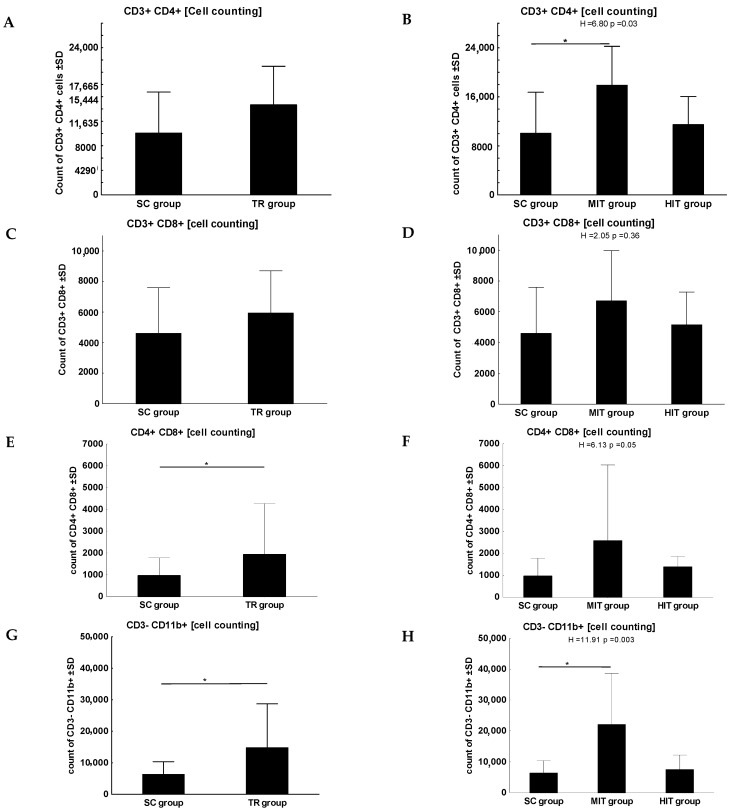

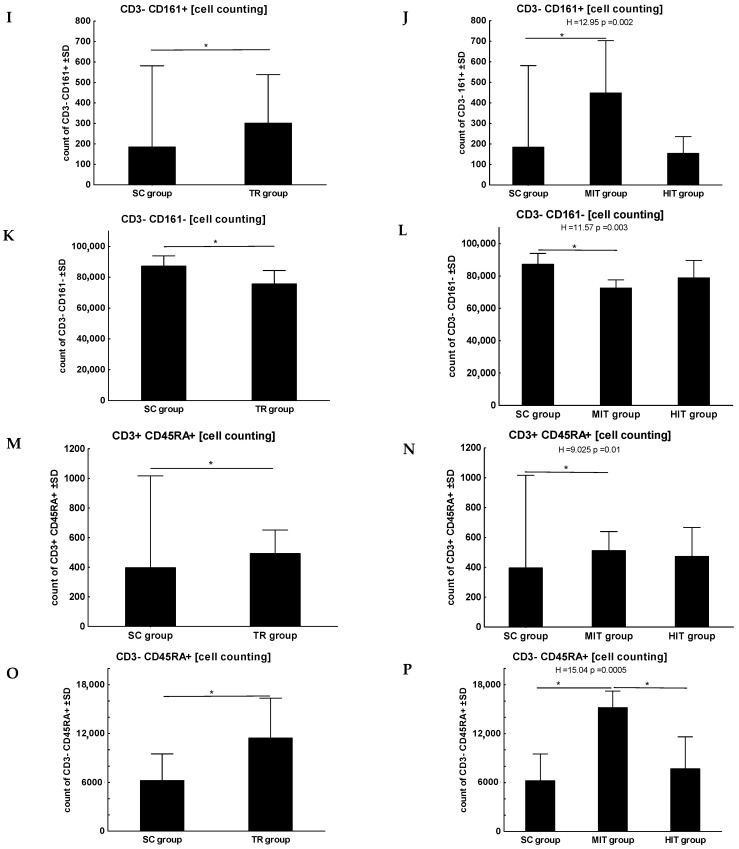
Characteristics of lymphocyte subpopulation: CD3+/CD4+ (**A**,**B**), CD3+/CD8+ (**C**,**D**), CD4+/CD8+ (**E**,**F**), CD3−/CD11b+ (**G**,**H**), CD3−/CD161+ (**I**,**J**), D3−/CD161− (**K**,**L**), CD3+/CD45RA+ (**M**,**N**) and CD3−/CD45RA+ (**O**,**P**) in the study groups. Measurements included: cell counting; the results were expressed as a cell number. Data were analyzed using the Mann–Whitney U test to compare the sedentary group (SC, *n* = 14) with the combined training groups (TR = MIT + HIT, *n* = 24) and the Kruskal–Wallis test was applied for the analysis in particular groups (SC, *n* = 14, MIT, *n* = 12, HIT, *n* = 12). *—the level of divergence between groups (*p* < 0.05); Mann–Whitney U test, Kruskal–Wallis test (post hoc).

No statistically significant difference was found between the training group and SC for the lymphocyte subpopulation of CD3+/CD4+ (Figure 2A). However, a significant difference was observed with respect to the level of physical training, with the highest value in the MIT group (SC vs. MIT, *p* = 0.03, Figure 2B). No significant differences were found in the lymphocyte subpopulations of CD3+/CD8+ (Figure 2C,D). However, a statistically significant difference was observed for the lymphocyte subpopulation CD4+/CD8+ for both groups (training group vs. SC, *p* = 0.01, Figure 2E) and for the intensity of physical training (*p* = 0.05, Figure 2F).

A statistically significant difference was observed between SC and the training group for the following lymphocyte subpopulations: CD3−/CD11b+ (*p* = 0.02, Figure 2G), CD3−/CD161+ (*p* = 0.002, Figure 2I), CD3−/CD161− (*p* = 0.002, Figure 2K), CD3+/CD45RA+ (*p* = 0.003, Figure 2M) and CD3−/CD45RA+ (*p* = 0.005, Figure 2O) with the highest efficacy of MIT for the following lymphocyte subpopulations: CD3−/CD11b+ (SC vs. MIT, *p* < 0.001, Figure 2H), CD3−/CD161+ (SC vs. MIT, *p* = 0.002, Figure 2J), CD3−/CD161− (SC vs. MIT, *p* = 0.002, Figure 2L), CD3+/CD45RA+ (SC vs. MIT, *p* = 0.02, Figure 2N) and CD3−/CD45RA+ (SC vs. MIT, *p* < 0.001, MIT vs. HIT, *p* = 0.02; Figure 2P).

**Figure 3:**
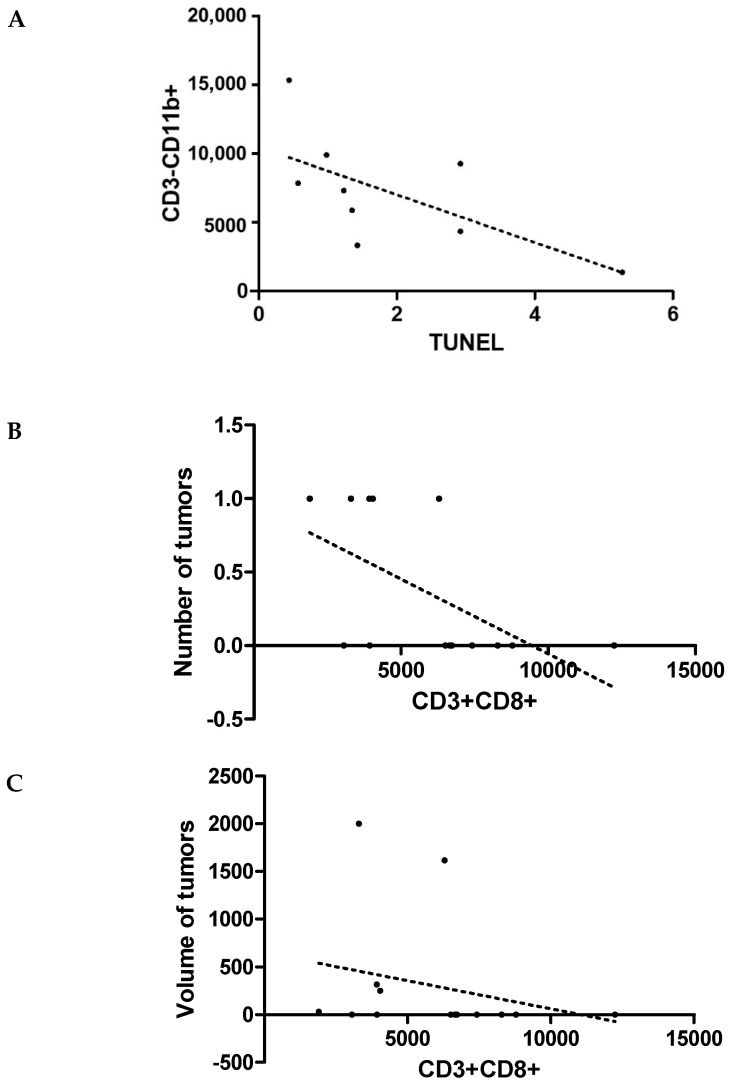
Analysis of Spearman rank correlations in the control group between the number of apoptotic cells and CD3−/CD11b (r = −0.76, *p* = 0.01, (**A**)) and in the training group, between the number of induced tumors and CD3+/CD8+ (r = −0.61, *p* = 0.02, (**B**)), and between their volume and CD3+/CD8+ (r = −0.56, *p* = 0.03, (**C**)).

Furthermore, negative correlations were observed in SC between the number of apoptotic cells and CD3−/CD11b (r = −0.76, *p* = 0.01, Figure 3A) and in the group of rats undergoing training between the number of induced tumors and CD3+/CD8+ (r = −0.61, *p* = 0.02, Figure 3B) and between their volume and CD3+/CD8+ (r = −0.56, *p* = 0.03, Figure 3C).

## 4. Discussion

The present study showed an increase in the number of specific lymphocyte pop-ulations (CD3+/CD4+, CD3−/CD11b+,CD3−/CD161+, CD3−/CD161−, CD3+/CD45RA+, CD3−/CD45RA+) when MIT was used. The available data indicate that exercise and regular physical activity have a significant modulating effect on immune function. In their review of the experimental, clinical, and epidemiological literature, Pedersen and Hoffman-Goetz [22] showed an increase in lymphocyte subpopulations during exercise (CD4+ T cells, CD8+ T cells, CD19+ B cells, CD16+ NK cells, CD56+ NK cells). Kalicki et al. [23] observed the effect of exercise on the number of B cells, which was over 20% higher in all exercised rat groups and Guo et al. [24] demonstrated positive results of exercise on improved immunity and T-cell function. Many studies have confirmed that, in trained animals, exercise adaptations comprise systemic alterations with improved immune function, reduced systemic inflammation, and improved metabolic health [25].

In this study, a significant inverse correlation was also observed between the number of tumors and their volume and the level of cytotoxic T cells (CD3+/CD8+). A reduction in the tumor size with an increase in NK cells and macrophages under the influence of physical training in mice was also observed by Pedersen and Hoffman-Goetz [22]. In their research, Pedersen et al. showed a similar relationship. NK cell infiltration was significantly increased in tumors from running mice, whereas the depletion of NK cells enhanced tumor growth and blunted the beneficial effects of exercise. In addition, the exercise also increased CD8+ and CD4+ cells [26]. The activity of CD8+ T lymphocytes seems to be best understood. Rundqvist et al. [27] showed that activated murine CD8+ T cells changed their central carbon metabolism in response to exertion in vivo, and immune cells from mice undergoing physical training were more potent antitumor effector cells when transferred into tumor-bearing untrained mice. These findings showed that CD8+ T cells were metabolically altered due to exercise in a manner that acted to improve their antitumoral efficacy. 

T-cells are considered to play a crucial role in controlling tumor [28,29], are central to adaptive immune responses [30].

Depending on the conditions in the tumor microenvironment, the response and functional state of immune cells may alter dynamically and undergo change from effector activity directed against tumor cells to regulatory or even suppressor activity that passively or actively supports tumor development [31].

Exercise can modulate the tumor microenvironment [32]. Preclinical studies have demonstrated that tumor perfusion and oxygenation increase from the very first exercise session. Since the tumor vasculature is immature and does not have myogenic and autonomic regulation, any transient increase in mean arterial pressure, as reported during exercise, results in an increase in tumor perfusion. Beyond the acute increase in perfusion, exercise training causes vessel normalization and enhances tumor blood flow and oxygenation in subsequent resting conditions. As a result, the tumor microenvironment is less hypoxic, making tumors less aggressive. This may partly account for the association of exercise training with reduced tumor burden and may indicate exercise training as a form of vascular normalization therapy. Of note, a normalized tumor vasculature and microenvironment reprograms the immunosuppressive tumor microenvironment and enhances antitumor immunity [33].

The increase in CD8+ T cells and NK cells in response to exercise are phenotypically effector and important for antitumor activity [26]. NK cells are the most responsive cells to the exercise-dependent mobilization, followed by CD8 T cells [34]. 

It is also interesting to note the exosomes from cancer cells that might also be associated with intracellular communications involved in the development of the tumor microenvironment. There have been several papers on the association between CD63 and cancer. It has been shown that exercise can attenuate the expression of exosome CD63 [35], which stimulates T-cells [30] and is presented as a prognostic marker for cancer [36]. Limited T-cell recruitment and function in the tumor microenvironment correlate with a poor prognosis in breast cancer [33].

The literature review also showed the evaluation of other lymphocyte subpopulations. An important relationship between exercise and the immune system (levels of Treg lymphocytes) and the reduction in tumor size was shown by Goh et al. [37] and Hagar et al. [38]. Physical training during cancer can help decrease Treg cell number and immunosuppressive effects. 

Also, DeMarzo et al. [39] showed an increase in the lymphocyte count and their activity in peripheral blood when physical activity was increased in a group of Wistar rats. The rapid and brief accumulation of leukocytes in peripheral blood during and immediately after physical activity was remarkably reproducible. While NK cells and CD8+ cytotoxic T cells seemed to be the leukocyte subsets with the largest increase, other populations, such as granulocytes, monocytes and B cells, also increased to varying degrees [40]. Similar observations were reported in our study.

## 5. Conclusions

Physical training, and MIT in particular, affected immune cell function (increase in NK, B lymphocytes, monocytes, T lymphocytes, Th lymphocytes, granulocytes, monocytes). Therefore, it can be used as a tool to optimize the immune system.

An altered immune response can be considered a mechanism underlying the effect of exercise on breast cancer development. In the physical training group, Tc lymphocytes influenced the number of tumors and their volume, which confirms their role in the development of cancer.

## Figures and Tables

**Table 2:** Lymphocyte subpopulations.

CD3+/CD4+	Th lymphocytes
CD3+/CD8+	Tc lymphocytes
CD4+/CD8+	double-positive immature lymphocytes
CD3−/CD11b+	granulocytes, monocytes
CD3−/CD161+	NK
CD3−/CD161−	B lymphocytes, monocytes
CD3+/CD45RA+	T lymphocytes
CD3−/CD45RA+	B lymphocytes, monocytes

## Data Availability

Data available on request.
